# Pragmatic evaluation of the Go2Play Active Play intervention on physical activity and fundamental movement skills in children

**DOI:** 10.1016/j.pmedr.2017.05.002

**Published:** 2017-05-22

**Authors:** Avril Johnstone, Adrienne R. Hughes, Xanne Janssen, John J. Reilly

**Affiliations:** University of Strathclyde, Scotland

**Keywords:** Active Play, Fundamental movement skills, Moderate to vigorous physical activity, Physical activity

## Abstract

Active play is a novel approach to addressing low physical activity levels and fundamental movement skills (FMS) in children. This study aimed to determine if a new school-based, ‘Go2Play Active Play’ intervention improved school day physical activity and FMS. This was a pragmatic evaluation conducted in Scotland during 2015–16. Participants (*n* = 172; mean age = 7 years) were recruited from seven primary schools taking part in the 5-month intervention, plus 24 participants not receiving the intervention were recruited to act as a comparison group.189 participants had physical activity measured using an Actigraph GT3X accelerometer at baseline and again at follow-up 5 months later. A sub-sample of participants from the intervention (*n* = 102) and comparison (*n* = 21) groups had their FMS assessed using the Test of Gross Motor Development (TGMD-2) at baseline and follow-up. Changes in school day physical activity and FMS variables were examined using repeated measures ANOVA. The main effect was ‘group’ on ‘time’ from baseline to follow-up. Results indicated there was a significant interaction for mean counts per minute and percent time in sedentary behavior, light intensity physical activity and moderate to vigorous physical activity (MVPA) (all *p* < 0.01) for school day physical activity. There was a significant interaction for gross motor quotient (GMQ) score (*p* = 0.02) and percentile (*p* = 0.04), locomotor skills score and percentile (both *p* = 0.02), but no significant interaction for object control skills score (*p* = 0.1) and percentile (*p* = 0.3). The Go2Play Active Play intervention may be a promising way of improving physical activity and FMS but this needs to be confirmed in an RCT.

## Highlights

• Active play is a promising way of increasing children's physical activity levels.
• Active play is a promising way of improving children's fundamental movement skills.
• Active play generates high levels of moderate to vigorous physical activity.
• Active play emphasises fun, which is key to engaging children in physical activity.

## 1 Introduction

Systematic reviews have provided high-quality evidence to support the role of physical activity in childhood, more specifically moderate to vigorous physical activity (MVPA), on improving health-related behaviors such as weight management; risks of cardiovascular disease, type 2 diabetes and high blood pressure (Janssen and Leblanc, 2010, Timmons et al., 2012). However, most children in western societies are not reaching the recommended 60 min of MVPA per day, with serious consequences on their health in later life (Department of Health, 2011, Basterfield et al., 2008; Healthy Behaviours in School Children (HBSC), 2015; Reilly et al., 2016a). A recent study by Reilly and colleagues suggested that children's physical activity levels decline at five years of age, approximately around the time they begin school (Reilly, 2016).

One neglected area of research is the possible role of active play in increasing children's physical activity. Active play involves children using large muscle groups to expend energy in physical activity which is unstructured, freely chosen and fun (Truelove et al., 2016). It has the potential for population-wide gains in habitual physical activity and MVPA levels if engagement is increased (Janssen, 2014, Tremblay et al., 2014).

Active play often takes place in outdoor settings, and outdoor time is associated with increased habitual physical activity and MVPA levels compared to time spent indoors (Cooper et al., 2010, Gray et al., 2015, King et al., 2011). However, contemporary children are engaging in less outdoor active play, probably due to parental safety concerns and the increasing use of screen-based activities (Veitch et al., 2006, Marshall et al., 2006). Active play may generate higher levels of MVPA compared to other domains of physical activity such as physical education (PE), recess, active transportation and other sports and physical activities, which have been the subject of more research effort (Hollis et al., 2016, Martin et al., 2016, Brazendale et al., 2015, Brockman et al., 2010, Reilly et al., 2016b).

Recent intervention studies have also suggested that active play may improve fundamental movement skills (FMS) (Jones et al., 2011, Adamo et al., 2016, Lai et al., 2014). FMS are important, as they are associated with increased physical activity and MVPA levels; however, FMS are typically poor in contemporary children (Lubans et al., 2010, Fisher et al., 2005a, Hardy et al., 2012, O'Brien et al., 2015). Therefore, facilitated active play sessions may be required for children to increase their physical activity levels and improve their FMS.

A school setting provides an ideal opportunity to influence children's physical activity levels and FMS (Lai et al., 2014, Dobbins et al., 2009). Schools have access to all children, including those from at-risk groups, who would otherwise not attend a community-based intervention (Story et al., 2009). A new school-based intervention called ‘Go2Play Active Play’ was facilitated by play workers, delivered weekly and lasted one-hour in duration. It used a combination of free play and active play to increase children's physical activity levels and improve their FMS. Therefore, the primary aim of this research was to determine if participation in the Go2Play Active Play intervention improved (a) school day physical activity and (b) FMS. A secondary aim was to estimate the intensity of activity during the Go2Play Active Play intervention compared to traditional PE in a comparison group.

## 2 Methods

### 2.1 Study design and participants

**Fig. 1:**
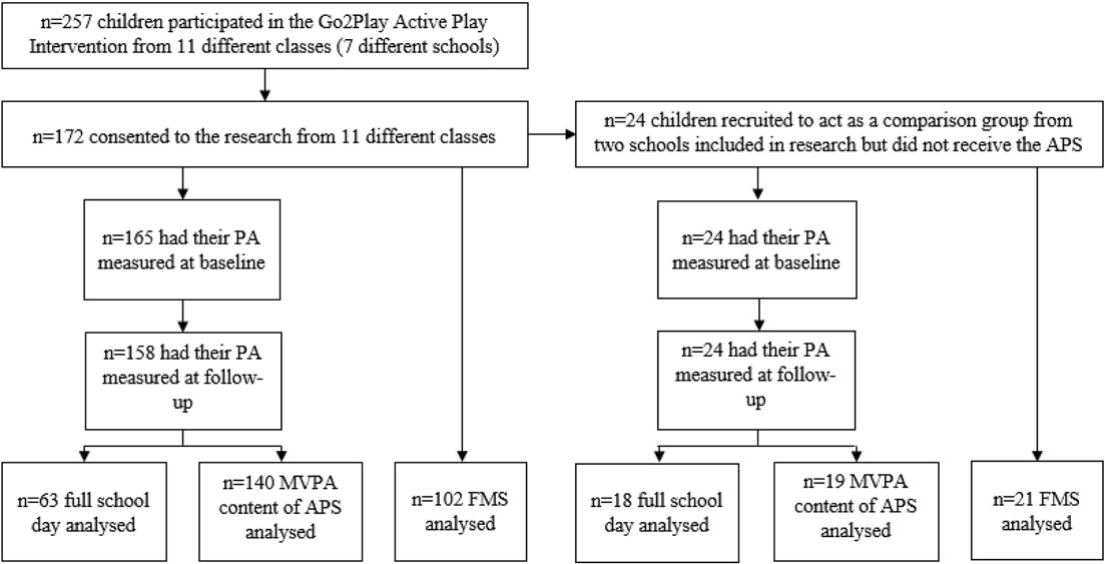
Presents a flow diagram to highlight the participants involved in the evaluation, number recruited and number analysed for each of the variables. Abbreviations: PA = physical activity, MVPA, moderate to vigorous physical activity, FMS = fundamental movement skills, APS = Active Play session, PE = physical education.

Fig. 1 presents an overview of the recruitment process and data analysed. This study was a 5-month pragmatic evaluation of a new school-based Go2Play Active Play intervention, in which data were collected at baseline during September and October 2015 and again at follow-up during February and March 2016. Seasonal effects were not likely to affect physical activity during data collection in this study as these have found to be small in Scotland (Fisher et al., 2005b).

Children (*n* = 257) from seven primary schools (involving eleven classes from primary grades 1–5) participated in the intervention. A total of 172 children (mean age = 7.0 years; SD = 1.1) provided written consent (via their primary care giver) to participate in the evaluation. Children were eligible for the evaluation if they were apparently healthy and able to participate in normal school activities.

Two of the schools already participating in the evaluation offered an additional two classes, who did not receive the Go2Play Active Play intervention, to act as the comparison group. A total of 24 children (from two classes; primary grades 2–4) provided consent via their primary care giver.

All schools participating in the present study were located in the west of Scotland where children's enrollment is based on area of residence. The consenting participants' demographics are presented in Table 1. Ethical approval was granted by the University of Strathclyde's School of Psychological Sciences and Health Ethics Committee prior to data collection.

**Table 1:** Demographics of consenting participants.

	Intervention (*n*=172)	Comparison (*n*=172)	Differences between baseline variables
	Mean (SD) or *n* (%)	Mean (SD) or *n* (%)	*p*-Value
Male	82 (48%)	8 (33%)	0.2
Female	90 (52%)	16 (67%)	
Age (years)	7.0 (1.1)	7.4 (0.9)	0.09
BMI z-score	0.4 (1.2)	0.7 (1.2)	0.3
*n* (%) living in top 15% most socio-economically deprived areas of Scotland	130 (76%)	20 (83%)	0.4

### 2.2 Pragmatic evaluation

The present study was considered from the planning stage to be a pragmatic evaluation. A pragmatic evaluation involves conducting research in ‘real world’ scenarios where decisions need to be made on how to best conduct the evaluation with the limited amount of time and resources the researchers may have. In relation to the present study, this meant that we could not control when the intervention began, the number of schools involved or how many Go2Play Active Play sessions and PE classes children engaged in at either baseline or follow-up. We were also unable to randomise schools or classes to the intervention or comparison group. Recruitment of the comparison group was based on convenience sampling as two schools already participating in the intervention offered an additional two classes who did not participate in Go2Play Active Play. Participants were similar in age, BMI z-score and socio-economic status (see Table 1).

### 2.3 Procedure

Once consent was provided, 189 participants (165 = intervention; 24 = comparison) were asked to wear an ActiGraph GT3X accelerometer for four school days (09:00–15:00) during September and October 2015. Due to a lack of time and resources, it was not possible to assess FMS of all consenting children, therefore a sub-sample of 123 children (102 = intervention; 21 = comparison) were randomly selected from the seven schools to have their FMS assessed using the Test of Gross Motor Development (TGMD-2) (Ulrich, 2000). Most children in the subsample had their baseline FMS assessed within one month of the intervention beginning. The participants in the intervention group continued their participation in the Go2Play Active Play intervention (comparison group continued their usual course of PE). At 5-months, the intervention and comparison groups had their physical activity and FMS re-assessed just before the intervention finished during February and March 2016. FMS were not assessed while participants were wearing their ActiGraph accelerometer as the FMS assessment may have affected their physical activity levels The mean duration at which FMS was measured at baseline and follow-up was 4 months (SD = 0.4).

### 2.4 Intervention

Agile CIC (www.agilecic.com) and Inspiring Scotland (www.inspiringscotland.org.uk) designed the Go2Play Active Play intervention collaboratively and conducted pilot work in 2014 before the independent evaluation began in 2015. The Go2Play Active Play intervention was underpinned by Whitehead's concept of physical literacy (Whitehead, 2001). Physical literacy is the development of physical competencies, motivation and confidence to be physically active throughout an individual's lifespan (Whitehead, 2001). Key to developing physical literacy and therefore increasing physical activity levels is creating an environment that fosters an enjoyment of physical activity from an early age while developing key movement skills. Evidence has suggested that active play achieves both enjoyment and development of FMS thus providing an evidence-based justification as to why active play was the type of physical activity selected for the intervention (Jones et al., 2011, Adamo et al., 2016).

The Go2Play Active Play intervention was outdoors, lasted one-hour in duration, was facilitated by local play workers (trained by Agile CIC), and combined structured games and free play (30 min each). The first half of the session aimed to introduce children to a variety of FMS by delivering fun, inclusive and active games focussed on improving a specific FMS area (for example locomotor or object control). Each session focussed on one FMS area so that a broad range of skills were covered over the 5-month intervention period. For example, if the first half of the session focused on object control, the play workers would facilitate games to develop children's catching or throwing ability (examples of the games played can be found at www.activeplay.org.uk). The second half was free play, which allowed children to practise what they learned in the first half of the session and/or to create and play their own games using a variety of traditional equipment such as balls, beanbags, cones, hoops etc. Additional information on the Go2Play Active Play programme can be found at www.inspiringscotland.org.uk/our-funds/go2play.

During the intervention, four classes participated in two; one-hour Go2Play Active Play sessions per week and the remaining seven classes participated in one, one-hour session per week for 5-months. The comparison group participated in their usual PE classes (described in Table 2).

**Table 2:** Overview of Active Play and PE sessions included in the measurement of *school day physical activity* at baseline and follow-up in the intervention and comparison groups.

School	Class	Number of children	Baseline		Follow-up	
			Number of Go2Play Active Play sessions	Number of PE classes	Number of Go2Play Active Play sessions	Number of PE classes
*Intervention (n = 63)*						
A	1	8	0	2 × 1 h	2 × 1 h	0
	2	25	0	2 × 1 h	2 × 1 h	1 × 1 h
B	3	20	0	2 × 50 min	2 × 1 h	0
	4	10	0	1 × 50 min	2 × 1 h	0

*Comparison (n = 18)*						
A	5	10	0	1 × 1 h	0	1 × 50 min
B	6	8	0	1 × 1 h	0	1 × 40 min, 1 × 1 h

### 2.5 Anthropometrics

All consenting participants had their height and weight measured (to the nearest 0.1 cm/kg) using a portable stadiometer and digital scales (both Seca, Hamburg, Germany). Weight status is presented as a BMI z-score relative to 1990 UK reference data; healthy weight (BMI z-score < 1.04); overweight (BMI z-score 1.04–1.64); obese (BMI z-score > 1.64). Postcode data were collected to describe the participant's area-based socio-economic status (SES) using the Scottish Index of Multiple Deprivation (SIMD) (The Scottish Government, 2016).

### 2.6 Physical activity

Participants wore an ActiGraph GT3X accelerometer (Pensacola, Florida, USA) for four school days (09:00–15:00), attached to an elastic waist belt and worn around the participant's waist so that the accelerometer was on or slightly above their right hip (Evenson et al., 2008). It was not feasible to measure physical activity during the after-school period. Data were collected in 15-s epochs and converted into total volume of physical activity (counts per minute, cpm) and physical activity intensities using cut points suggested by Evenson and colleagues, which have evidence of reliability and validity (Evenson et al., 2008). These cut points are sedentary behavior (0–100 cpm), light intensity physical activity (101–2292 cpm), moderate intensity physical activity (2293–4008 cpm) and vigorous intensity physical activity (> 4008 cpm).

#### 2.6.1 School day

Data were accepted if the participants wore the monitor for a minimum of three school days (09:00–15:00) and if school-day physical activity was measured before the intervention started (*n* = 63). Evidence suggests a minimum wear-time of three days for 6 h/day has acceptable reliability (Basterfield et al., 2011); in the present study, children wore the accelerometer on average for 4 days for 6 h/day (09:00–15:00) at baseline and follow-up. Intervention participants meeting the above criteria (*n* = 63) were from two schools (four classes, primary 2–4) and were compared to the comparison group (*n* = 18) who were recruited from the same two schools, but did not receive the intervention (two classes, primary 2–4). Variables analysed were percent time in sedentary behavior, light intensity physical activity and MVPA. Table 2 describes the duration and frequency of Go2Play Active Play and PE sessions engaged in during the measurement week at baseline and follow-up.

#### 2.6.2 Go2Play Active Play sessions

Go2Play Active Play sessions and PE sessions (for the comparison group) were extracted from the participants' follow-up physical activity data. Participants in the intervention group were included in the data analysis if they participated in one full Go2Play Active Play session (*n* = 140) or one full PE class for the comparison group (*n* = 19) during the follow-up measurement week. If they participated in two Go2Play Active Play or PE sessions (for the comparison group) during the measurement week an average was taken. Variables analysed were counts per minute and percent time in sedentary behavior, light intensity physical activity and MVPA to correct for the different duration of the PE and Go2Play Active Play sessions.

### 2.7 FMS

FMS were measured by the same field staff and researcher at baseline and follow-up using the TGMD-2, which is a valid, reliable and cost-effective method for assessing FMS (Wiart and Darrah, 2001). The researcher trained field staff prior to data collection according to the TGMD-2 manual. They were given practise opportunities to administer and score the test with children to ensure they were competent at measuring FMS.

The TGMD-2 assesses 12 skills and is split into two subtests; locomotor (run, gallop, hop, leap, horizontal jump, slide) and object control (strike, dribble, catch, throw, kick, roll). Each of the 12 skills is divided into a number of components that make up the skill. For the assessment, the field staff demonstrated the skill first, and then the child performed the skill twice and was observed and scored accordingly (Ulrich, 2000). If the child being assessed completed the component of the skill as written in the TGMD-2 manual they scored, a ‘1’ and a ‘0’ if they did not.

Participants were included in the data analysis if they had their FMS assessed at both baseline and follow-up: 102 children in the intervention group and 21 children in the comparison group (total *n* = 123). Variables examined were gross motor quotient (GMQ) score and percentiles, which is a summary score of all FMS that adjusts for age and gender and is the recommended variable for interpretation as it is the most reliable indicator of FMS competency (Ulrich, 2000). Standard scores and percentiles were also used for interpretation of each subtest (locomotor and object control), which are not as reliable as the GMQ score but are a useful interpretation of both subtests (Ulrich, 2000).

### 2.8 Data analysis

All statistical analyses were conducted using SPSS v 22.0 (SPSS Inc., Chicago, IL). Tests for normality were run prior to data analysis to check for normal data distribution (skewness and kurtosis <|2.0 |). Descriptive statistics were run to present means and standard deviations for relevant variables for both physical activity and FMS. Baseline differences in demographics, physical activity and FMS variables between the intervention and comparison group were assessed using an independent samples *t*-tests, chi square test or Mann Whitney *U* test (demographic differences are presented in Table 1). The two primary aims of improvement in FMS variables and school day physical activity were examined using repeated measures ANOVA. The main effect was ‘group’ (intervention and comparison) on ‘time’ from baseline to follow-up.

## 3 Results

### 3.1 Objectively measured physical activity

#### 3.1.1 School day physical activity

At baseline, the intervention and comparison group were similar in percent time in sedentary behavior and light physical activity but the comparison group had a higher mean counts per minute (*p* = 0.03) and percent time in MVPA (*p* = 0.02). Table 3 presents the changes in school day physical activity from baseline to follow-up in the intervention and comparison group.

**Table 3:** School day physical activity at baseline and follow-up in intervention and comparison groups (changes are presented as an average day).

	Intervention (*n* = 63)				Comparison (*n* = 18)			
	Baseline	Follow-up	Mean change (95% CI)	*p*-Value	Baseline	Follow-up	Mean change (95% CI)	*p*-Value
Counts per minute	610 (137)	868 (180)	258 (217 to 300)	< 0.01	741 (220)	676 (164)	− 65 (142 to 13)	0.1
Sedentary time (%)	52.2 (5.9)	33.6 (11.6)	− 18.6 (− 21.2 to − 16.0)	< 0.01	49.5 (7.9)	49.5 (12.6)	0.1 (− 4.8 to 4.9)	1.0
Light PA (%)	39.8 (5.0)	55.5 (11.7)	15.7 (13.0 to 18.5)	< 0.01	39.8 (5.5)	41.6 (12.1)	1.7 (− 3.4 to 6.9)	0.5
MVPA (%)	8.0 (2.6)	10.8 (4.0)	2.8 (1.9 to 3.7)	< 0.01	10.7 (4.3)	8.9 (2.5)	− 1.8 (− 3.5 to − 0.1)	0.04

Data presented as mean (SD). Abbreviations: PA = Physical Activity, MVPA = Moderate to Vigorous Physical Activity.

There was a significant interaction between ‘time’ and ‘group’ for mean counts per minute (*F*(1,79) = 53.9, *p* < 0.01) and percent time in: sedentary behavior (*F*(1,79) = 45.3, *p* < 0.01), light intensity physical activity (*F*(1,79) = 22.6, *p* < 0.01) and MVPA (*F*(1,79) = 23.0, *p* < 0.01).

The intervention group showed a decrease in percent time in sedentary behavior (− 18.6%), an increase in total physical activity (+ 258 cpm) and percent time in light intensity physical activity (+ 15.7%) and MVPA (+ 2.8%, *p* < 0.01 for all). The comparison group showed a decrease in mean counts per minute (− 65 cpm, *p* = 0.1), an increase in percent time: in sedentary behavior (0.1%, *p* = 1.0) and light physical activity (1.7%, *p* = 0.5), and a decrease in percent time in MVPA (− 1.8%, *p* = 0.04).

#### 3.1.2 Intensity of physical activity during Go2Play Active Play and PE sessions

Means and standard deviations for the intensity of physical activity during Go2Play Active Play for the intervention group and PE for the comparison group are presented in Table 4.

**Table 4:** Intensity of physical activity during Active Play sessions and PE in intervention and control groups.

	Intervention (*n* = 140)	Comparison (*n* = 19)
Counts per minute	1716 (523)	1314 (381)
Sedentary time (%)	19.1 (12.2)	33.2 (8.1)
Light PA (%)	50.8 (12.7)	45.8 (7.7)
MVPA (%)	30.1 (12.4)	21.1 (7.2)

Data presented as mean (SD). Abbreviations: PA = physical activity, MVPA = moderate to vigorous physical activity.

### 3.2 FMS

At baseline, the intervention and comparison group were similar in all FMS variables. Table 5 presents the changes in FMS variables from baseline to follow-up in the intervention and comparison group.

**Table 5:** FMS at baseline and follow-up in intervention and comparison groups.

	Intervention (*n* = 102)				Comparison (*n* = 21)			
	Baseline	Follow-up	Mean change (95% CI)	*p*-Value	Baseline	Follow-up	Mean change (95% CI)	*p*-Value
GMQ score	83.2 (11.6)	93.3 (11.1)	10.1 (7.9 to 12.3)	< 0.01	86.6 (11.2)	90.1 (10.9)	3.6 (− 1.3 to 8.4)	0.15
GMQ percentile	18.9 (17.8)	36.1 (23.8)	17.2 (13.2 to 21.2)	< 0.01	23.4 (19.8)	30.2 (20.3)	6.9 (− 2.0 to 15.7)	0.13
Locomotor score	7.5 (2.1)	9.1 (2.4)	1.6 (1.1 to 2.1)	< 0.01	7.5 (1.6)	7.8 (1.6)	0.3 (− 0.7 to 1.3)	0.59
Locomotor percentile	24.6 (18.8)	40.4 (25.5)	15.9 (11.1 to 20.6)	< 0.01	23.0 (13.7)	25.6 (14.9)	2.5 (− 8.0 to 13.0)	0.64
Object control score	6.9 (2.4)	8.7 (2.1)	1.8 (1.3 to 2.3)	< 0.01	8.0 (2.7)	9.0 (2.4)	0.9 (− 0.1 to 1.9)	0.08
Object control percentile	21.5 (20.0)	36.7 (23.3)	15.2 (10.7 to 19.7)	< 0.01	30.0 (25.9)	39.9 (25.2)	9.9 (0.0 to 19.7)	0.05

Data presented as mean (SD); GMQ, gross motor quotient.

#### 3.2.1 GMQ

There was a significant interaction between ‘time’ and ‘group’ for GMQ score (*F*(1,121) = 5.9, *p* = 0.02) and GMQ percentile (*F*(1,121) = 4.4, *p* = 0.04).

The pairwise post hoc comparison indicated that the intervention group had a statistically significant increase in both their GMQ score and their GMQ percentile (both *p* < 0.01). In the comparison group, there was an increase in the GMQ score (*p* = 0.15) and GMQ percentile (*p* = 0.13), but neither were statistically significant.

#### 3.2.2 Locomotor and object control skills

There was a significant interaction between ‘time’ and ‘group’ for locomotor skills score (*F*(1,121) = 5.4, *p* = 0.02) and locomotor percentile (*F*(1,121) = 5.2, *p* = 0.02. There was no significant interaction between ‘time’ and ‘group’ for object control skills score (*F*(1,121) = 2.5, *p* = 0.1) and object control percentile (*F*(1,121) = 0.9, *p* = 0.3).

The pairwise post hoc comparison indicated that the intervention group had a statistically significant increase in their locomotor skills score and percentile and their object control skills score and percentile (all *p* < 0.01). The comparison group's locomotor skills score (*p* = 0.59) and percentile (*p* = 0.64), and their object control skills score (*p* = 0.08) and percentile (*p* = 0.05) also increased, but the increases were not statistically significant.

## 4 Discussion

The present study suggested that a 5-month Go2Play Active Play intervention significantly improved physical activity and FMS variables compared to the comparison group, who received their usual PE. However, since this was a pragmatic evaluation, it was not possible to randomly allocate classes to intervention and comparison groups and the size of the comparison group was small.

Recent research has suggested that children's physical activity levels decline around the age they start school (Reilly, 2016). School hours are often very physically inactive periods of the day; and therefore, a critical time where improvements need to be made (van Stralen et al., 2014, Nettlefold et al., 2011, Belton et al., 2016). Much of the research aimed at increasing physical activity levels during school has focussed on PE, recess and active transportation, all of which have shown limited improvements (Hollis et al., 2016, Martin et al., 2016, Reilly et al., 2016b). School-based interventions utilising active play are limited and tend to focus on recess interventions (Reilly et al., 2016b, Verstraete et al., 2006). These studies have shown limited improvements compared to the findings in the present study where percent time spent in light physical activity and MVPA during the school day improved by 15.7% and 2.8%, respectively.

After-school is an important period of the day where children engage in even less physical activity than during school hours (Brockman et al., 2010, Belton et al., 2016). Although the present study only focussed on the effect of the intervention during the school day, it highlights the need to objectively measure physical activity after school to determine the true effect of the intervention. The influence on physical activity may be greater in the present study because children are learning to play with limited involvement from adults, and equipment that is readily available in most homes.

It is thought that active play has the potential to generate higher levels of MVPA compared to other types of physical activity (Janssen, 2014, Brazendale et al., 2015). In the present study, children spent, on average, 30.1% of the Go2Play Active Play session in MVPA compared to the comparison group who spent 21.1% of their PE class in MVPA. Brazendale and colleagues found the MVPA content of an hour of free play was 35%, which is similar to Go2Play Active Play (Brazendale et al., 2015). International recommendations suggest that children should spend 50% of their time in MVPA during PE (Association for Physical Education, 2008). Although the MVPA content of Go2Play Active Play sessions did not achieve the 50% recommended time in MVPA, it appears that active play in the present study may generate higher levels of MVPA compared to traditional PE.

FMS need to be improved as they are low in children from western nations and are associated with increased physical activity and MVPA levels (Fisher et al., 2005a, Hardy et al., 2012). Interventions aimed at improving children's FMS have been successful in a range of settings including, early years, school and community-based studies (Logan et al., 2012). Two school-based interventions, which focused on sports, provided improvements in some FMS skills but in general, the overall improvements in these studies were small compared to the present study (Lai et al., 2014, Barnett et al., 2009, Salmon et al., 2008). However, recent interventions that utilised active play to improve FMS have shown improvements in pre-school aged children and are more consistent with findings in the present study (Jones et al., 2011, Adamo et al., 2016). The mean GMQ score at baseline in our study was 83.2 (18.9th percentile) and significantly improved to 93.3 (36.1st percentile) in the intervention group. These scores, even at follow-up, are lower than the norm-referenced value of 100 presented by Ulrich (Ulrich, 2000). In fact, it is widely thought that FMS are generally poor in contemporary children and worse in those with low socioeconomic status (Hardy et al., 2012, O'Brien et al., 2015). In the present study, 76% of the participants in the intervention group were from Scotland's most socio-economically deprived areas. While the present study had some limitations, discussed below, it tentatively suggests that the Go2Play Active Play intervention may be effective improving FMS. The mix of facilitated FMS games and child-led free play may create an environment that fosters natural curiosity in a child to practise FMS by themselves in an enjoyable way.

The present study was a pragmatic evaluation of a school-based active play intervention delivered by three local charities in central Scotland. Despite potentially promising findings, and this study being a novel attempt to evaluate an active play intervention as a means of improving both FMS and physical activity, it had some important limitations. Firstly, this study was a pragmatic evaluation meaning certain important elements of study design were out with the researcher's control. These included: when the Go2Play Active Play intervention began, the number of schools who participated in the intervention and how many active play sessions and PE classes they engaged in at both baseline and follow-up. The sample size was determined by the number of participating schools; therefore, a power calculation was not carried out and our ability to detect any change in the comparison group (e.g. in FMS) was probably limited due to the small number of children in this group. Second, the schools could not be randomised to the intervention or control group as schools were already selected before the research was underway. Third, the effect of active play on habitual physical activity (i.e. including time spent out of school) needs further exploration to determine the true potential of active play on increasing overall physical activity. Results obtained should be helpful in developing a randomised controlled trial (RCT) to provide a more definitive evaluation of Go2Play Active Play in the future.

## Conflicts of interest

None.

## Role of funding source

This work was supported by Inspiring Scotland; 502 Gorgie Road, Edinburgh, EH11 3AF (www.inspiringscotland.org.uk). Inspiring Scotland are a Company Limited by Guarantee registered in Scotland, No. SC342436, and a registered Scottish charity, No. SC039605. The funders were not involved in any aspect of the research.

## References

[bb0005] Adamo K.B., Wilson S., Harvey A.L. (2016). Does intervening in childcare settings impact fundamental movement skill development?. Med. Sci. Sports Exerc..

[bb0010] Association for Physical Education (2008). http://www.afpe.org.uk/physical-education/wp-content/uploads/afPE_Health_Position_Paper_Web_Version2015.pdf.

[bb0015] Barnett L.M., van Beurden E., Morgan P.J., Brooks L.O., Zask A., Beard J.R. (2009). Six year follow-up of students who participated in a school-based physical activity intervention: a longitudinal cohort study. Int. J. Behav. Nutr. Phys. Act..

[bb0020] Basterfield L., Adamson A.J., Parkinson K.N. (2008). Surveillance of physical activity in the UK is flawed: validation of the Health Survey for England Physical Activity Questionnaire. Arch. Dis. Child..

[bb0025] Basterfield L., Adamson A.J., Pearce M.S., Reilly J.J. (2011). Stability of habitual physical activity and sedentary behavior monitoring by accelerometry in 6- to 8-year-olds. J. Phys. Act. Health.

[bb0030] Belton S., O'Brien W., Issartel J., McGrane B., Powell D. (2016). Where does the time go? Patterns of physical activity in adolescent youth. J. Sci. Med. Sport.

[bb0035] Brazendale K., Chandler J.L., Beets M.W. (2015). Maximizing children's physical activity using the LET US Play principles. Prev. Med..

[bb0040] Brockman R., Jago R., Fox K.R. (2010). The contribution of active play to the physical activity of primary school children. Prev. Med..

[bb0045] Cooper A.R., Page A.S., Wheeler B.W., Hillsdon M., Griew P., Jago R. (2010). Patterns of GPS measured time outdoors after school and objective physical activity in English children: the PEACH project. Int. J. Behav. Nutr. Phys. Act..

[bb0050] Department of Health (2011). StartActive, Stay Active. https://www.gov.uk/government/uploads/system/uploads/attachment_data/file/216370/dh_128210.pdf.

[bb0055] Dobbins M., De Corby K., Robeson P., Husson H., Tirilis D. (2009). School-based physical activity programs for promoting physical activity and fitness in children and adolescents aged 6–18. Cochrane Database Syst. Rev..

[bb0060] Evenson K.R., Catellier D.J., Gill K., Ondrak K.S., McMurray R.G. (2008). Calibration of two objective measures of physical activity for children. J. Sports Sci..

[bb0065] Fisher A., Reilly J.J., Kelly L.A. (2005). Fundamental movement skills and habitual physical activity in young children. Med. Sci. Sports Exerc..

[bb0070] Fisher A., Reilly J., Montgomery C. (2005). Seasonality in physical activity and sedentary behavior in young children. Pediatr. Exerc. Sci..

[bb0075] Gray C., Gibbons R., Larouche R. (2015). What is the relationship between outdoor time and physical activity, sedentary behaviour, and physical fitness in children? A systematic review. Int. J. Environ. Res. Public Health.

[bb0080] Hardy L.L., Reinten-Reynolds T., Espinel P., Zask A., Okely A.D. (2012). Prevalence and correlates of low fundamental movement skill competency in children. Pediatrics.

[bb0085] Healthy Behaviours in School Children (HBSC) (2015). Findings From the HBSC 2014 Survey in Scotland. http://www.cahru.org/content/03-publications/04-reports/hbsc_nr14_interactive_final.pdf.

[bb0090] Hollis J.L., Williams A.J., Sutherland R. (2016). A systematic review and meta-analysis of moderate-to-vigorous physical activity levels in elementary school physical education lessons. Prev. Med..

[bb0095] Janssen I. (2014). Active play: an important physical activity strategy in the fight against childhood obesity. Can. J. Public Health.

[bb0100] Janssen I., Leblanc A.G. (2010). Systematic review of the health benefits of physical activity and fitness in school-aged children and youth. Int. J. Behav. Nutr. Phys. Act..

[bb0105] Jones R.A., Riethmuller A., Hesketh K., Trezise J., Batterham M., Okely A.D. (2011). Promoting fundamental movement skill development and physical activity in early childhood settings: a cluster randomized controlled trial. Pediatr. Exerc. Sci..

[bb0110] King A.C., Parkinson K.N., Adamson A.J. (2011). Correlates of objectively measured physical activity and sedentary behaviour in English children. Eur. J. Pub. Health.

[bb0115] Lai S.K., Costigan S.A., Morgan P.J. (2014). Do school-based interventions focusing on physical activity, fitness, or fundamental movement skill competency produce a sustained impact in these outcomes in children and adolescents? A systematic review of follow-up studies. Sports Med..

[bb0120] Logan S.W., Robinson L.E., Wilson A.E., Lucas W.A. (2012). Getting the fundamentals of movement: a meta-analysis of the effectiveness of motor skill interventions in children. Child Care Health Dev..

[bb0125] Lubans D.R., Morgan P.J., Cliff D.P., Barnett L.M., Okely A.D. (2010). Fundamental movement skills in children and adolescents: review of associated health benefits. Sports Med..

[bb0130] Marshall S.J., Gorely T., Biddle S.J. (2006). A descriptive epidemiology of screen-based media use in youth: a review and critique. J. Adolesc..

[bb0135] Martin A., Boyle J., Corlett F., Kelly P., Reilly J.J. (2016). Contribution of walking to school to individual and population moderate-vigorous intensity physical activity: systematic review and meta-analysis. Pediatr. Exerc. Sci..

[bb0140] Nettlefold L., McKay H.A., Warburton D.E., McGuire K.A., Bredin S.S., Naylor P.J. (2011). The challenge of low physical activity during the school day: at recess, lunch and in physical education. Br. J. Sports Med..

[bb0145] O'Brien W., Belton S., Issartel J. (2015). Fundamental movement skill proficiency amongst adolescent youth. Phys. Educ. Sport Pedagog..

[bb0150] Reilly J.J. (2016). When does it all go wrong? Longitudinal studies of changes in moderate-to-vigorous-intensity physical activity across childhood and adolescence. J. Exerc. Sci. Fit..

[bb0155] Reilly J.J., Johnstone A., McNeill G., Hughes A.R. (2016 Nov). Results from Scotland's 2016 report card on physical activity for children and youth. J. Phys. Act. Health.

[bb0160] Reilly J.J., Johnston G., McIntosh S., Martin A. (2016). Contribution of school recess to daily physical activity: systematic review and evidence appraisal. Health Behav. Policy Rev..

[bb0165] Salmon J., Ball K., Hume C., Booth M., Crawford D. (2008). Outcomes of a group-randomized trial to prevent excess weight gain, reduce screen behaviours and promote physical activity in 10-year-old children: switch-play. Int. J. Obes..

[bb0170] Story M., Nanney M.S., Schwartz M.B. (2009). Schools and obesity prevention: creating school environments and policies to promote healthy eating and physical activity. Milbank Q..

[bb0175] van Stralen M.M., Yıldırım M., Wulp A. (2014). Measured sedentary time and physical activity during the school day of European 10- to 12-year-old children: the ENERGY project. J. Sci. Med. Sport.

[bb0180] The Scottish Government (2016). The Scottish Index of Multiple Deprivation. http://www.gov.scot/Topics/Statistics/SIMD.

[bb0185] Timmons B.W., Leblanc A.G., Carson V. (2012). Systematic review of physical activity and health in the early years (aged 0–4 years). Appl. Physiol. Nutr. Metab..

[bb0190] Tremblay M.S., Gray C.E., Akinroye K. (2014). Physical activity of children: a global matrix of grades comparing 15 countries. J. Phys. Act. Health.

[bb0195] Truelove S., Vanderloo L.M., Tucker P. (2016). Defining and measuring active play among young children: a systematic review. J. Phys. Act. Health.

[bb0200] Ulrich D.A. (2000). Test of Gross Motor Development-2.

[bb0205] Veitch J., Bagley S., Ball K., Salmon J. (2006). Where do children usually play? A qualitative study of parents' perceptions of influences on children's active free-play. Health Place.

[bb0210] Verstraete S.J., Cardon G.M., De Clercq D.L., De Bourdeaudhuij I.M. (2006). Increasing children's physical activity levels during recess periods in elementary schools: the effects of providing game equipment. Eur. J. Pub. Health.

[bb0215] Whitehead M. (2001). The concept of physical literacy. Eur. J. Phys. Educ..

[bb0220] Wiart L., Darrah J. (2001). Review of four tests of gross motor development. Dev. Med. Child Neurol..

